# Preventing blood transfusion failures: FMEA, an effective assessment method

**DOI:** 10.1186/s12913-017-2380-3

**Published:** 2017-06-30

**Authors:** Zhila Najafpour, Mojtaba Hasoumi, Faranak Behzadi, Efat Mohamadi, Mohamadreza Jafary, Morteza Saeedi

**Affiliations:** 1 Health Care Management, Department of Health Economics and Management, School of Public Health, Students’ Scientific Research Center, Tehran University of Medical Sciences, Tehran, Iran; 2 Health Economics, Health Management and Economics Research Center, Iran University of Medical Sciences, Tehran, Iran; 3 Health Policy, Department of Health Economics and Management, School of Public Health, Tehran University of Medical Sciences, Tehran, Iran; 4 Shariati Hospital, Tehran University of Medical Sciences, Tehran, Iran; 5 Emergency Medicine research center, Shariati hospital, Tehran University of Medical Sciences, Tehran, Iran

**Keywords:** Blood transfusion, Failure modes, Risk analysis, FMEA

## Abstract

**Background:**

Failure Mode and Effect Analysis (FMEA) is a method used to assess the risk of failures and harms to patients during the medical process and to identify the associated clinical issues. The aim of this study was to conduct an assessment of blood transfusion process in a teaching general hospital, using FMEA as the method.

**Methods:**

A structured FMEA was recruited in our study performed in 2014, and corrective actions were implemented and re-evaluated after 6 months. Sixteen 2-h sessions were held to perform FMEA in the blood transfusion process, including five steps: establishing the context, selecting team members, analysis of the processes, hazard analysis, and developing a risk reduction protocol for blood transfusion.

**Results:**

Failure modes with the highest risk priority numbers (RPNs) were identified. The overall RPN scores ranged from 5 to 100 among which, four failure modes were associated with RPNs over 75. The data analysis indicated that failures with the highest RPNs were: labelling (RPN: 100), transfusion of blood or the component (RPN: 100), patient identification (RPN: 80) and sampling (RPN: 75).

**Conclusion:**

The results demonstrated that mis-transfusion of blood or blood component is the most important error, which can lead to serious morbidity or mortality. Provision of training to the personnel on blood transfusion, knowledge raising on hazards and appropriate preventative measures, as well as developing standard safety guidelines are essential, and must be implemented during all steps of blood and blood component transfusion.

## Background

The first report of the Institute of Medicine (IOM) entitled “To Err Is Human” has caused medical errors to be considered as a major priority worldwide [1]. The findings from studies on medical errors occurring in blood transfusion practices disclose that 3.7% of patients in the U. S are vulnerable to adverse events during hospital stay [2, 3]. An adverse event is defined as an unintended injury or complication with the severity ranging from very little to fatal consequences [4].

Error may occur during all therapeutic procedures among which blood transfusion is one of the most common high risk procedures. Since transfusion errors may result in serious morbidity or mortality, they are categorized as *critical medical errors*. Ordinarily much attention is paid to the safety of the blood products prior to transfusion, and not necessarily to the processes involved in the actual blood transfusion at bedside [5] where approximately 70% of the errors have been reported to occur [6]. Therefore, in every step of blood transfusion there are potential risks, such as errors in patient identification, blood typing, cross-matching and other human errors. There are various methods available for risk analysis and management to enhance the quality of care and patient safety, among which one can name [7] probabilistic risk assessment (or fault tree analysis), root cause analysis, reporting systems, and failure mode and effect analysis (FMEA) [8]. The latter method is used to assess the risk of failures in blood transfusion and identifies other critical obstacles. While FMEA was first applied in the aerospace industry in the 1960s, public health care systems initiated to make use of this method in 1990s, particularly in case of high risk processes such as manufacturing drugs and preventing medication errors.

Although FMEA is time-consuming and requires organizational commitment, it is efficient for the identification and prioritization of potential risks during blood transfusion practices [9]. Upon our recent review of literature, there are few published studies available investigating FMEA application in blood transfusion. Nevertheless, blood transfusion has not been examined comprehensively in various clinical settings except for few studies reporting the risks of certain sub-processes of blood transfusion [10, 11]. Further, the incidence and risks involved in blood transfusion may differ depending on the clinical setting, supporting the need for examining blood transfusion processes specific to each institution. In this vein, our study design is differed from others since we assessed the blood transfusion processes pre- and post-intervention. Also, given our own clinical setting and infrastructure, our results could be different from those reported by earlier studies pinpointing to the fact that blood transfusion processes should be examined and/or revised at individual basis. Despite numerous studies conducted worldwide on identifying and preventing blood transfusion errors with the use of FMEA, uncertainties remain regarding ways of improving the blood transfusion safety in Iran, due to dearth of research. The aim of this prospectively designed study was to identify the potential hazards involved in blood transfusion and to quantify their effects (by RPN score) after taking the risk reduction measures into account so that recommendations would be provided for improving the processes under our investigation using FMEA method.

## Methods

Our study was conducted at a teaching general hospital affiliated to Tehran University of Medical Sciences in June 2014. Failure Mode and Effect Analysis (FMEA), certain corrective measures, and audits over a 6-month period were used in this study to fulfill the objectives. Also, 16 sessions (2-h each) were held to perform FMEA in blood transfusion process. In this study we performed FMEA in five steps consisting of: a) establishing the context, b) selecting team members, c) analysis of the processes d) hazard analysis and e) developing a risk reduction protocol for blood transfusion.

### Establishing the context

The risk reduction protocol was developed upon the review of every step involved, from physician requisition to the actual blood transfusion.

### Selecting team members

The analysis of blood transfusion and identification of the associated risks were conducted by a 12-member team of health professionals, including physicians, nurses, and staff from various disciplines such as information technology, quality control, clinical laboratory and blood bank. Also, a staff from Patient Safety Department served as the team coordinator and consultant. Members of the team attended a one-day training workshop for orientation to FMEA.

### Analysis of the processes

As listed below the steps involved in blood transfusion were reviewed in detail through brainstorming, interviews and taking notes during direct observations:

i. Registering physician requisition for blood and/or blood components was made on special forms.

- Indications for blood and/or blood product transfusion were as follows: *Platelets:* < 10 × 109/L Pl; *RBC:* acute blood loss of greater than 1500 mL or 30 percent of blood volume; *Hemoglobin:* an Hb count of < 7–8 g/dL depending on the condition; *FFP:* an INR ≥ 2.0 or ≥ 1.5 for neurosurgical patients [12].

ii. Taking blood from the patients and dispatching them to the blood bank.
iii. Processing and interpreting each requisition by a blood bank technician.
iv. Preparing and delivering the requested blood orders to the hospital wards.
v. Transfusing the ordered blood and/or blood product by a nurse.

### Hazard analysis

To identify potential risks, the failures or hazards possible for every step was listed by each of the team members, while assigning their individual score to each failure. The assigned scores were frequency of occurrence, detecting failures, effects before occurrence and severity of the effects, described in the Hazard Scoring Matrix [13] (Table 1). Scores were multiplied for each potential failure and the overall risk priority number (RPN) was determined by calculating the mean of all RPNs assigned to each failure by the team members. RPN or critical index is a quantitative expression for the evaluation of each failure. Subsequently, the lowest and highest possible RPNs were ranged from 1 (1×1×1) as the “best” score to 125 (5×5×5) as the “worst” one.

**Table 1:** Hazard Scoring Matrix

Rating	Severity of Hazard (S)	Occurrence of Hazard (O)	Detectability of Hazard (D)
1	No injury, or patient monitoring Only	Rare – Failure unlikely to occur (happens in 1 out of 10,000 observed episodes).	Always detected: Detected 9/10 times
2	Temporary injury with need of additional interventions or treatments	Low – Relatively few failures (happen in 1 out of 1000 observed episodes).	Probably detected: 7/10 times
3	Temporary injury with increased length of hospital stay or increased level of care	Moderate – Occasional failures (happen in 1 out of 200 observed episodes).	middle probability of detection: detected 5/10 times
4	Permanent lessening of body function	Often – Repeated failures (happen in 1 out of 100 observed episodes).	Probably undetected (low): detected 2/10 times
5	Death or permanent loss of major functions	Always– Commonly occurring failure (happens in 1 out of 20 observed episodes).	Undetected: 0/10 times

Failures were detected based on the Priority Matrix, which helped the team to decide upon which items to focus and work on, as well as for analysing failure mode causes, making recommendations, and planning new control measures to eliminate or reduce risk. Prioritization was made based on RPNs, consisting of four different areas requiring priority interventions as follows: 1 = Emergency (the highest), 2 = Urgent, 3 = Programming and control, and 4 = Monitoring (the lowest); (see Table 2). Failures that exceeded the acceptable limits (i.e. the highest RPN) were identified, prioritized, and used for developing the risk reduction protocol. We considered additional factors for corrective actions such as consequences of a failure (including impact on patient and confidential, peer-review process), available resources, and hospital policies and practices.

**Table 2:** Application of risk analysis (severity score against occurrence and detection) in blood transfusion process (Risk matrix*: O – D - S)

S^a^	1	2	3	4	5
O^b^ × D^c^					
5	5	10	15	20	25
10	10	20	30	40	50
15	15	30	45	60	75
20	20	40	60	80	100
25	25	50	75	100	125

The RPN or Risk matrix is calculated by multiplying the severity^a^ (S), occurrence^b^ (O), and detectability^c^ (D) of the hazard. For example, a hazard that has “major severity” (rate 5), that occurs “often” (rate 4), and that has a “low probability of detection” (rate 4) has an RPN of 5 × 4 × 4 = 80

Score more than 80 = emergency for intervention (eliminated)

Score 40–75 = urgent for intervention (programing & controlled)

Score 20–30 = need to program (programing & controlled)

Score less than 20= need to monitor (accepted)

### Developing a risk reduction protocol

Followed by ranking, the team focused on failures that scored higher than 75, as listed in Table 2 and suggested a set of corrective measures for consideration by the hospital administration and ultimate implementation.

The list of corrective measures was termed as “*Plan, Do, Check, Act* (PDCA)”. The “*Plan*” represents the identification and analysis of potential failures. The “*Do*” appears for developing potential solutions. The “*Check*” ensures testing the efficacy of each measure, and the “*Act*” appears for the timely and complete implementation of the corrective measures.

If a measure was ineffective in clinical practice, another PDCA measure would be introduced until the desired end point was achieved. Accordingly, the FMEA method was repeatedly evaluated and the RPN scores were revised until optimal outcomes were achieved.

A good example of revision of the existing procedures was the automated labelling and barcoding of blood samples. To achieve the desired outcomes, the responsible staffs were trained and their performance was monitored which led to a significant improvement in patient safety. Over the 6-month period, the potential failures and the corrective measures were regularly reassessed, leading to the development of the current risk reduction plan that had been practiced and mandated at the hospital since the team presented its final corrective measures for blood transfusion based on FMEA.

## Result

During the analysis, all blood transfusion steps were detected based on direct observations and related experts’ opinions. Potential failures and possible causes were identified in the blood transfusion process. A total of 31 failures were identified with RPN scores ranging from 2 to 100 (Table 3). The S, O, and D for each risk were calculated. Four blood transfusion failures were identified with RPNs over 75 (Table 4), for which we identified the causes and recommended the appropriate risk reduction measures. Our analyses indicated that the failure risks with the highest RPNs were blood sample labelling (RPN 100), incorrect order for blood or the component transfusion (RPN: 100), error in patient identification (RPN: 80), and sampling (RPN: 75). The overall RPN ranged from a minimum of 5 to a maximum of 100 (see Table 4).

**Table 3:** Evaluation of Risk Priority Numbers (RPNs) including the Severity Score, the Probability of Occurrence Score, and the Probability of Detection Score and related corrective action for highest RPN – Steps1–3

Step	Failure mode	Possible cause	(O)	(D)	(S)	RPN	Action
Ordering blood request	- Inappropriate ordering (kind and amount) of blood/blood component* - Ordering transfusion of blood products for a patient who does not need blood transfusion or vice versa - Overuse or underuse of blood products - Physician use of fresh frozen plasma instead of Cryoprecipitate for controlling blood dyscrasia	- Inappropriate assessment of patient’s clinical need for blood, and when required. - not being able to check that the ordered product is the most suitable with regard to diagnosis	3	2	1	6	-
	- Blood plasma abuse	Blood plasma still used in volume expansion, as nutritional supplement and to improve immunoglobulin levels	1	2	1	2	
	Incorrectly recorded patient characteristics	- Look alike patient names	1	4	4	16	
	Incomplete filling of the request form	- New patient orders into a record from a prior encounter	5	3	3	45	
	- incorrect or incomplete entry of the patient’s data	- Environment factors	1	5	4	20	
	- Incorrect entry of results in the database of the Laboratory Information System.	- Request form filled out incorrectly/incompletely	1	5	4	20	
	- Inappropriate assessment of patient’s clinical need for blood, and when required.	Staff negativity Heavy workload Low level of literacy patients	3	2	1	6	
Patient identification	-Incorrect patient identification	- Patient’s with same name - Time constraints	2	5	5	50	- Using identification devices e.g. Bracelets with an alphanumeric code, Machine-readable bracelets with barcodes or radiofrequency identifier devices (RFID), Machine-readable anthropometric data.
	-Not Checking ID Bracelet	- Unaware - Non adherence to guideline, assumption that -admission process and/or assessment by doctor has confirmed identity	4	5	4	80	
Blood taking from patient (sampling)	-Taking blood from the wrong patient	- Mismatched information on specimen and requisition - Not Check ID wristbands of patient - Non adherence to guideline, workload (multiple patients to attend to) or incorrect patient documents taken to bedside - Unlabeled tubes taken from bedside and cluttered workspace which multiple staff use causes confusion - Technical error in sampling by nursing staff	3	5	5	75	- Training and frequent education should be provided to phlebotomy staff responsible for drawing blood bank specimens. - Hand labeling of specimens at the patient’s bedside should be mandatory - Clinical staff given training courses on blood transfusion - Allocate phlebotomist personnel - Sign in phlebotomist’s name on sample tube
Steps 4 and 5							
attaching labels on tubes, and Sending the samples and request form	- Labels attached to tube at nursing station	- Non-use from scanners and label printers for patient ID and specimen labeling	4	5	5	100	- Reject potentially mislabeled or misidentified specimens - Safe environment - Automated systems for patient identification and sample labeling - Strict adherence to standard guidelines and recommendations for specimen collection - Service staff given training courses on transport blood bag and sample of patient
	- Labeling tubes some patients simultaneously	- inadequate equipment - lack of policy procedure	4	5	5	100	
	- Pre written labels at work station	- Lack of supervision and continuing education	4	5	5	100	
	- Mismatch between the labels on the tubes and the request form	- Designing of Process of Blood Collection - Non-compliance with the cold chain	4	4	3	80	
analysis of blood samples	- Incorrect blood group	- Non-use of Guidelines	1	5	5	25	
	- Incorrect typing, technical error	- Knowledge deficit	2	5	2	20	
	- Incorrect typing, clerical error	- Lack of supervision and continues monitoring	2	5	2	20	
	Inaccurate cross-matching	- Inexpert staff	1	5	5	25	
	- Incorrect specimen used for testing	- No calibration equipment	2	4	2	16	
	Non Checking of sample and request Form	- User error	1	1	5	5	
	Not Verifying blood type with historical type of patient	- Misinterpretation	1	5	1	5	
Steps 6 and 7							
Preparing and delivering blood bag	- Errors in Record the identification patient	- Inexpert staff - Poorly visible labels and request form - Unsafe technique when preparing blood - Knowledge deficit	1	5	3	15	
	- Wrong product - Wrong blood bag being sent from blood bank ward	- Inefficient blood delivery system - Inadequate staff and equipment	1	5	3	15	
	- Delivered blood bag to the wrong unit	- Untimely communication - Human factors - Heavy workload at the blood bank - A large number of blood samples have to be delivered to different departments at the same time - Waiting for an elevator, limited staff for delivering blood	1	5	5	25	
Transfusing blood components	No Double-checking at bedside with patient’s ID band by two nurses and sign their names	- Human factors - Knowledge deficit - Heavy workload in ward	4	2	4	32	- Training on the signs and symptoms of transfusion reactions - Training about how to initiate early intervention, and submitting specimens and materials - Necessary for completing a transfusion workup - Protocol should mandate close monitoring of the patient during the first 10 to 20 min of transfusion. - Development accountability and special attention to problems of transfusion and reporting errors
	Not verifying the type, quality, amount, and kind of the blood taken from the blood bank ward	Knowledge deficit - Lack of standard procedure	3	3	4	36	
	- Failure to monitoring for signs in the first 15 min	- Lack of standard procedure - Knowledge deficit	5	3	5	75	
	- preparation time before infusion of more than 30 min and non-checked Vital signs every 30 min until the transfusion is complete	- Patient not informed to alert practitioners to treat symptoms - Non Complete records - The transfusion doesn’t complete within 4 to 6 h	5	3	5	75	
	- Treat condition/not educated patient	- The final patient identification check at the bedside not performed by nurses - Inappropriate transfusion time	5	5	4	100	
	Failure to comply with the standard conditions in the maintenance of blood in ward		3	2	4	24	
	- Blood transfusion reaction occurring during the transfusion process and non suitable control by nurse		1	3	3	9

**Table 4:** Prioritization of the highest RPNs, with Corresponding Proposed Actions to be implemented to avoid the Occurrence of the Individual Failure Modes

Phase	before implementation of precautions (RPN score)	action	Major Recommendation	after implementation of precautions (RPN score)
Patient misidentification	80	Eliminated	Using identification devices e.g. Bracelets with an alphanumeric code, Machine-readable bracelets with barcodes	25
Missampling	75	Controlled	Training staff, Allocate phlebotomist personnel	20
Mislabeling	100	Eliminated	Training staff, Reject potentially mislabeled or misidentified specimens by blood department Automated systems for patient identification and sample labeling	30
Incorrect implementation of order for transfusing blood component	100	Eliminated	Formulated Protocol about Appropriateness and safety of blood transfusion and Training on the signs and symptoms of transfusion reactions and about how to initiate early intervention, and submitting specimens and materials	30

Based on the risk assessment, action plans were determined to reduce the risks of these four failure modes. The recommended risk reduction measures, as well as the RPNs for the failure modes implemented before and after the precautions are presented in Table 4. Followed by implementing the preventive measures against these risks over a period of 6 months, rescoring was performed by the previous team. This revealed a reduction in the value of all PRNs assigned to potential failures so that RPN scores of patient mis-identification, mislabelling, incorrect order for blood/component transfusion and mis-sampling decreased to 25, 30, 30 and 20, respectively (Table 4). All measures implemented were therefore retained and continued. No adverse events such as sample collection from wrong patient and ABO-incompatible transfusion were detected since the implementation of preventive measures.

## Discussion

Blood transfusion is a major therapeutic process where appropriate risk management leads to significant improvement in the quality of services and patient safety. We applied FMEA method to analyse the risks of blood transfusion in a hospital. We focused not only on the actual blood transfusion, but also on labelling, sampling, patient identification, and other factors that may be associated with errors in blood transfusion procedures.

In this study, blood transfusion failures with the highest RPN score had their roots in poor team working, miscommunication, and lack of modern and standard equipment. On the other hand, blood transfusion failures with low RPN scores stemmed from the institutional procedures and the nature of the staff training. Our multidisciplinary team provided recommendations for controlling and improving the blood transfusion failures.

Errors in blood transfusion practices can be very critical and are related to one or more procedural steps throughout physician requisition to the actual blood transfusion [14]. Our results indicated that incorrect implementation of order for transfusing blood component was the most important step, leading to serious morbidity or mortality. Further errors may occur under the following circumstances: *a)* failure to comply with standard procedures for blood maintenance *b)* shortfall in completion of blood transfusion within 4 h of the requisition, *c)* lack of monitoring of the patient’s signs and symptoms at least during the first 15 min of the transfusion, and *d)* poor or lack of monitoring of reactions, such as fever, hypotension, vomiting, diarrhea, discolored urine, apnoea, or collapse. These issues can be avoided if the staff are well-trained and retrained periodically. Also, patients themselves can help reduce or even prevent the incidence of transfusion failures, if they are instructed and warned about the reaction signs and symptoms.

To address transfusion issues, this study recommends three corrective actions a) developing procedures for submitting specimens, b) nursing staff attendance in blood transfusion workshops, and c) developing a standard protocol for monitoring patients during the first 15 min from blood transfusion and for monitoring signs and symptoms of transfusion reactions.

In this study, other failures with high RPN scores were related to sampling and labelling of blood tubes, consistent with reports from previous studies [15, 16]. These could occur due to the unclear or lack of standard procedures in patient identification and sample labelling. Consistently, a previous study [17] has reported that the incidence of labelling errors decreased significantly in the first year followed by replacing barcodes and modern computer technology for patient identification rather old equipments. Other causes of transfusion failures identified by this study included using out-dated techniques for blood sampling, incorrect labelling, loss of correct labels, or failure to check patient identification carefully prior to transfusion [12]. Also, we noted that lack of reliable patient identifiers and inability to correctly recognize patients were additional issues impacting patient identification. Other important factors affecting safe transfusion practices include lack of standard procedures, high workload and shortage of nursing staff for blood transfusion, and misunderstandings that may occur in the transfusion processes. Results reported by Makary et al. [18] shows that out of 1000 surgical specimens identification errors occurred in 4.3 cases and a total of 182 mislabelled specimens occurred on an annual basis. Lippi’s study [19] has reported that misidentification of general laboratory specimens is around 1% and can cause serious harm to patients.

To prevent errors in patient identification, this study greatly emphasizes the use of two identifiers for patient identification. We also recommend using modern information technology for entering patient data, automated systems and barcoding for specimen labelling and patient identification. We equally point up that the nursing staff should be trained periodically on the use of reliable methods for identifying blood samples, checking patient medical records and their identification. On this basis, staff knowledge needs to be updated on important precautionary measures relevant to safe patient identification. Lastly, assigning more staff to blood transfusion and reducing the hours of assignment to avoid fatigue can greatly reduce patient misidentifications and ultimately improves blood transfusion processes [20].

In this study, transfusion practices resulting in ‘wrong blood in a tube’ occurred as follows: marking blood samples away from the bedside, failure to check patient identity, using prewritten labels, blood sampling by staff lacking phlebotomy training and/or experience. To prevent such errors, it is recommended that sheets of labels should not be separated from the patients’ chart, tubes should not be taken away from the bedsides and patients’ identification must always be carried out. Fortunately, most sampling errors can be detected and rectified by the laboratory based on their record of the patients [21]. We have addressed effective ways to avoid these problems in the action plans we developed and introduced (Table 4).

In this study, we used certified phlebotomists instead of nurses for drawing blood and sampling. We also, recommended new hospital policies to reduce transfusion errors and to ensure safe sampling and labelling. A good example was that blood samples must be taken from the arm not from the IV catheter connected to patients. It is also important that the phlebotomist complete the tube labelling before leaving the patient’s bedside and matching the identification details with the patient’s wristband.

Besides four failures discussed above, the authors of the present study have recognized non-technical (i.e., human) errors as being the most important set of factors in blood transfusion practices. Linden [22] reported that more than 50% of transfusion errors occurred outside the blood bank. This study indicates that blood was administered to wrong recipients in 38% of cases and phlebotomy errors happened in 13% of cases. The results also indicate that 15% of transfusion failures were due to multiple errors, the most common of which being failure to detect them at bedside. On the contrary, Ibojie [23] suggested that 95% of transfusion errors were due to poor compliance with the standard guidelines. We recommend developing specific clinical guidelines, monitoring system, and quality assurance program in order to effectively prevent non-technical errors involved in blood transfusion.

The study strength came from the ability to improve the existing processes for blood transfusion and the introduction of new procedures which significantly improved the safety and reduced the clinical risks in study setting. We also consider reliance on the views of a team of experts to discuss and analyse changes to the existing transfusion practices as a major strength of this study. The lack of standardization on how the failure modes should be prioritized can be considered as a limitation. In absence of an alternative protocol, we prioritized transfusion failures based on RPN scores and the available resources, policies and plans of the hospital where this study was conducted.

## Conclusion

Blood transfusion failures carry the potential risk of causing catastrophic consequences. Therefore, serious attention it requires. We identified factors that lead to blood transfusion failures in a general hospital setting. We also developed intervention strategies to prevent the failures. We found that among pre-analytical errors, misidentification represents a crucial failure prior to the blood analysis and involved serious risks to the patient. Since human factors have a pivotal role in preventing transfusion failures, we strongly recommend the provision of training to physicians, nurses and staff on the hazards, prevention, safety and compliance with technical guidelines involved in blood transfusion practices. The rate of blood transfusion failures declined after we implemented FMEA method and established the corrective interventions at the hospital. We conclude that this method should be observed to enhance the safety, reliability, risk evaluation, quality control and the blood transfusion processes.

## Abbreviations

D: Detectability
FMEA: Failure mode and effect analysis
IOM: Institute of medicine
O: Occurrence
PDCA: Plan, do, check, act
RPN: Risk priority number
S: Severity
